# ProBDNF as a Myokine in Skeletal Muscle Injury: Role in Inflammation and Potential for Therapeutic Modulation of p75^NTR^

**DOI:** 10.3390/ijms26010401

**Published:** 2025-01-05

**Authors:** Katherine Aby, Ryan Antony, Tao Yang, Frank M. Longo, Yifan Li

**Affiliations:** 1 Division of Basic Biomedical Sciences, University of South Dakota Sanford School of Medicine, Vermillion, SD 57069, USA; rantony@unmc.edu; 2 University of Nebraska Medical Center, Omaha, NE 68198, USA; 3 Department of Neurology and Neurological Sciences, Stanford University, Stanford, CA 94305, USA; yangt@stanford.edu (T.Y.); flongo@stanford.edu (F.M.L.); 4 Department of Basic Sciences, California Northstate University College of Medicine, Elk Grove, CA 95757, USA

**Keywords:** p75^NTR^, proBDNF, myokine, LM11A-31, skeletal muscle injury, ischemia–reperfusion, inflammation, splenic monocytes

## Abstract

Brain-derived neurotropic factor (BDNF) is expressed by skeletal muscle as a myokine. Our previous work showed that the active precursor, proBDNF, is the predominant form of BDNF expressed in skeletal muscle, and that following skeletal muscle injury, proBDNF levels are significantly increased. However, the function of the muscle-derived proBDNF in injury-induced inflammation has yet to be fully understood. Using a model of tourniquet-induced ischemia–reperfusion (IR) injury of the hindlimb, this study presents, for the first time, strong and novel evidence that following IR injury, proBDNF is released from skeletal muscle into circulation as an endocrine signaling molecule. Further, this study shows that 1 day post-IR injury, the proBDNF receptor, p75^NTR^, is upregulated 12-fold in splenic monocytes, which are known to be quickly mobilized to the injury site. We demonstrate that p75^NTR^ plays a role in the activation of splenic monocytes, and that treatment with a p75^NTR^ small-molecule modulator, LM11A-31, significantly reduced monocyte inflammatory responses upon lipopolysaccharide stimulation. Overall, the present study establishes proBDNF as a myokine that plays a significant role in skeletal muscle injury-induced inflammation through its receptor, p75^NTR^, which may be modulated using LM11A-31 as potential translational therapeutic against injury and inflammation.

## 1. Introduction

Skeletal muscle makes up nearly 40% of a human’s body weight [1,2]. Due to its size and location on the periphery of the body, it is at an increased risk of serious injury [3,4,5,6]. One prevalent injury type is skeletal muscle ischemia–reperfusion injury (IR) which often occurs as a result of trauma, peripheral vascular disease, surgery, or tourniquet application [7,8]. Skeletal muscle IR injuries occur when an acute decrease in blood flow is followed by the rapid return of blood flow [9]. This is often seen following the placement of a tourniquet in trauma situations or during limb surgeries to create a bloodless surgical field [7,8,10]. Skeletal muscle is unique in its ability to regenerate itself following skeletal muscle injury through a process of sterile inflammation, inflammation resolution, and skeletal muscle regeneration [11,12,13,14,15,16]. Many details, including the proteins and signaling involved in these processes, have yet to be fully understood, especially in tourniquet-induced IR injury.

A complex coordination of events is required in order for regeneration of skeletal muscle to occur. This relies not only on traditional well-established factors, but also on novel players that are continually being discovered. Among these novel players are a series of cytokines, growth factors, and other small molecules released from the injured skeletal muscle, which are termed myokines. Among the growing list of myokines is brain-derived neurotrophic factor (BDNF) [1,17,18]. Many studies on myokine BDNF have focused on the role of BDNF in skeletal muscle following exercise, after which it has been shown to be highly upregulated and to play a role in maintenance of the neuromuscular junction, skeletal muscle metabolism, and myogenesis [19]. The role of BDNF in skeletal muscle injury is largely unknown, however. Importantly, BDNF has an active precursor, proBDNF. Our previous study in mice and a study by Edman et al. in humans both indicate that proBDNF is the dominantly expressed form of BDNF in skeletal muscle, consistently showing levels above those of mature BDNF [20,21,22]. Furthermore, we observed that this upregulation of myokine proBDNF drove the proinflammatory process in a murine model of tourniquet-induced skeletal muscle IR injury [21]. P75^NTR^ is a receptor with high binding affinity for proBDNF and other pro-neurotrophins, including pro-nerve growth factor, pro-neurotrophin-3, and pro-neurotrophin-4 [23]. Our data showed that p75^NTR^ signaling was activated in denervation-induced and IR-induced muscle injury [20,21]. Recent studies suggest that p75^NTR^ is involved in neuroinflammation [24] as well as peripheral inflammation [25]. However, it remains unclear how proBDNF and p75^NTR^ modulate the proinflammatory process following skeletal muscle injury. Additionally, whether myokine proBDNF was released into circulation to participate in endocrine signaling to distant immune cells or other tissues was unknown, but this is an important consideration for the present study and the field of myokine research. The present study aims to address these questions.

One possible target cell population is the splenic monocyte reservoir. Beyond its traditional roles, the spleen has been shown to harbor a population of monocytes that are capable of being quickly mobilized following injury [26,27,28]. Additionally, it has been reported that splenic monocytes may be recruited through novel signaling pathways besides the well-known macrophage chemoattractant protein-1 (MCP-1)-driven recruitment of bone marrow-derived monocytes [28]. This, combined with data showing reduced infiltration of myeloid cells into the injured tissue when proBDNF levels were reduced, led us to test the hypothesis that proBDNF released from skeletal muscle may target splenic monocytes to promote cytokine release and/or the recruitment of the monocytes to the injury site.

## 2. Results

### 2.1. Serum proBDNF Is Increased Following IR Injury

**Figure 1:**
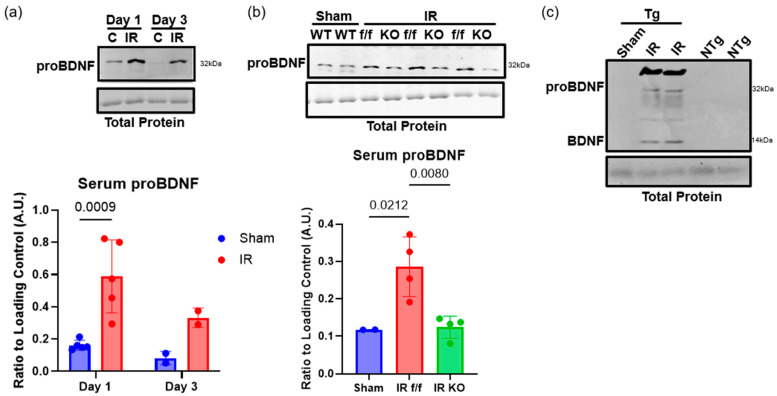
ProBDNF is dominantly expressed in and released from IR-injured skeletal muscle in a murine model. (**a**) Representative Western blots of proBDNF in skeletal muscle from contralateral control (C) muscle tissue and IR-injured muscle tissue on reperfusion days 1 and 3 and corresponding quantification; n = 2–5 analyzed using 2-way ANOVA followed by Tukey’s post hoc analysis, significance at *p* < 0.05. (**b**) ProBDNF in serum of uninjured sham wildtype (WT) animals or IR-injured BDNF floxed animals (f/f) or BDNF skeletal muscle knockout following IR injury on reperfusion day 3 and corresponding quantification; n = 2–4 analyzed using one-way ANOVA followed by Tukey’s post hoc analysis, significance at *p* < 0.05. (**c**) Western blot of serum for proBDNF and mature BDNF from sham and IR-injured transgenic MetRS* (Tg) animals and non-transgenic controls (NTg) following click chemistry and streptavidin pull down of biotinylated proteins. Error bars represent SD.

To begin, we tested whether the significant increase in proBDNF in skeletal muscle was released into circulation. Western blot was used to assess serum proBDNF levels in animals with IR injury compared to sham controls. The result revealed that animals with IR injury had a significant 1.8-fold increase in levels of serum proBDNF on reperfusion day 1, suggesting that the upregulation of proBDNF in the injured skeletal muscle is released into circulation (Figure 1a). To determine whether the increase in serum proBDNF was from skeletal muscle, BDNF flox controls and BDNF skeletal muscle knockout (skmKO) animals were subjected to IR injury and serum proBDNF levels were compared to those in sham animals. This revealed that proBDNF was significantly increased 2.4-fold in BDNF flox animals following IR injury compared to sham animals; however, in BDNF skmKO animals with hindlimb IR injury, serum proBDNF levels were similar to those of sham animals (Figure 1b). These data further suggest that increased serum proBDNF levels following IR injury are due to upregulation and release of proBDNF from skeletal muscle. To further confirm that the increased serum proBDNF originated from skeletal muscle, a mouse model of tissue specific protein labeling was used [29]. To this end, transgenic animals expressing Cre recombinase driven by the human alpha-actin striated promotor (HSA-Cre79) were crossed with animals expressing a modified tRNA synthetase. This allowed for skeletal muscle-specific expression of the modified tRNA synthetase. The transgenic animals were given the noncanonical amino acid azidoleucine (ANL) via IP injection. Only skeletal muscle tissue expressing the modified tRNA synthetase will be able to incorporate noncanonical ANL into nascent proteins. Any proteins released from skeletal muscle into circulations will be ANL-labeled proteins. Serum ANL-labeled proteins were pulled down and identified with click chemistry reaction, as detailed in the methods section. The transgenic animals and non-transgenic controls were subjected to tourniquet-induced hindlimb IR injury or a sham procedure and the serum was collected on reperfusion day 1. In the sham and IR-injured non-transgenic animals, no proBDNF was detected and pulled down; however, in the IR-injured transgenic animals, proBDNF was tagged and pulled down from the serum (Figure 1c). This is direct evidence showing that the increased levels of serum proBDNF in animals with IR injury are released from injured skeletal muscle.

### 2.2. BDNF Skeletal Muscle Knockout Reduces Inflammation and Regeneration Following Tourniquet-Induced IR Injury

**Figure 2:**
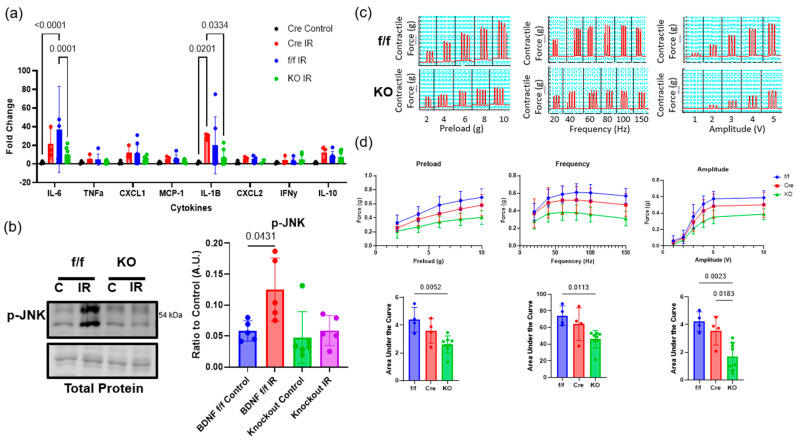
BDNF skeletal muscle knockout reduces inflammation and regeneration following tourniquet-induced IR injury. (**a**) Quantification of qPCR using log fold squared for cytokines in Cre Control, BDNF floxed control, and BDNF skeletal muscle knockout (KO) animals following IR injury; n = 3–7 analyzed using 2-way ANOVA followed by Tukey’s post hoc analysis, significance at *p* < 0.05. (**b**) Representative Western blots of phosphorylated (p) JNK in skeletal muscle from contralateral control (C) muscle tissue and IR-injured muscle tissue from BDNF flox (f/f) or BDNF skmKO (KO) animals and corresponding quantification; n = 5 analyzed using one-way ANOVA and Tukey’s post hoc test, significance at *p* < 0.05. (**c**) Representative preload, frequency, and amplitude force graphs for floxed control (f/f) and BDNF skeletal muscle knockout (KO) animals. (**d**) Quantification of preload, frequency, and amplitude in floxed control (f/f), HSA-Cre (Cre), and BDNF skeletal muscle knockout (KO) (top) and area under the curve analysis for preload, frequency, and amplitude (bottom); n = 4–8 analyzed using one-way ANOVA followed by Tukey’s post hoc test, significance at *p* < 0.05. Error bars represent SD.

Animals were subjected to hindlimb IR injury and inflammation in BDNF skmKO was compared to that in BDNF flox and HSA-Cre animals on reperfusion day 3 using qPCR. The qPCR results showed that proinflammatory cytokines IL-6 and IL-1β were significantly reduced in BDNF skmKO animals compared to BDNF wildtype and HSA-Cre animals, respectively (Figure 2a). Compared to BDNF wildtype animals, phosphorylation of c-Jun N-terminal kinase (JNK), a p75^NTR^ downstream signal, was significantly reduced in BDNF skmKO animals following IR injury (Figure 2b). This suggests that proBDNF, through p75^NTR^ signaling, drives the inflammatory phenotype in skeletal muscle following tourniquet-induced IR injury. In the reduced inflammatory environment, we wondered how this would impact regeneration and functional recovery. To test this, skeletal muscle contraction in BDNF floxed controls and BDNF skmKO animals were assessed on reperfusion day 12 using in situ skeletal muscle contraction with preload–force, frequency–force, and amplitude–force tests (Figure 2c,d). Despite muscle sizes being comparable, area under the curve analysis revealed that skeletal muscle of BDNF skmKO animals had significantly reduced performance in all three tests following IR injury (Figure 2c,d). Since acute inflammation is necessary to remove the damaged tissue and activate myogenesis, these functional results suggest that a reduction in proBDNF-driven inflammation in BDNF skmKO animals has negative effects on skeletal muscle regeneration and functional recovery following injury.

### 2.3. PAI-039 Treatment Reduces the Proinflammatory Phenotype

**Figure 3:**
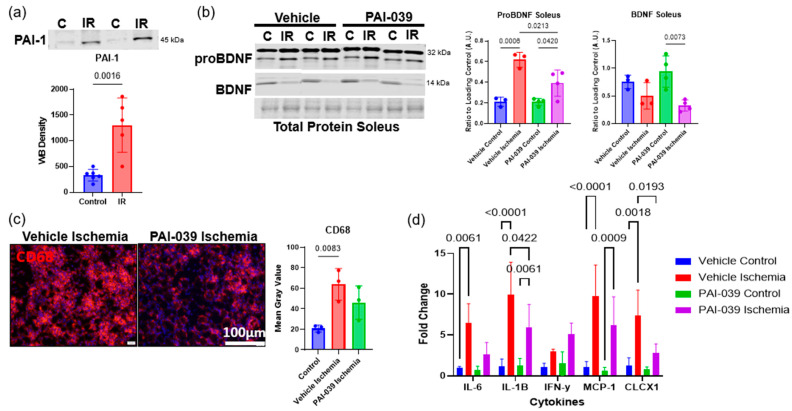
PAI-039 treatment reduces the proinflammatory phenotype. (**a**) Representative Western blots of PAI-1 in skeletal muscle from contralateral control (C) muscle tissue and IR-injured muscle tissue and corresponding quantification; n = 5–6 analyzed using Student’s *t* test, significance at *p* < 0.05. (**b**) Representative Western blots of proBDNF and BDNF in skeletal muscle of from contralateral control (C) muscle tissue and IR-injured muscle tissue in vehicle or PAI-039-treated animals and corresponding quantification; n = 3–4 analyzed using one-way ANOVA followed by Tukey’s post hoc analysis, significant at *p* < 0.05. (**c**) Representative images of immunofluorescent staining for CD68+ immune populations in vehicle and PAI-039-treated animals on reperfusion day 3 and corresponding quantification; n = 3 analyzed using one-way ANOVA followed by Tukey’s post hoc analysis, significance at *p* < 0.05. (**d**) Quantification of qPCR for cytokines in vehicle and PAI-039-treated animals following IR injury represented as log fold changes; n = 3–4 analyzed using 2-way ANOVA followed by Tukey’s post hoc analysis, significance at *p* < 0.05. Error bars represent SD.

We found that following tourniquet-induced IR injury, plasminogen activator inhibitor-1 (PAI-1), a negative regulator of the proBDNF cleaving protein tissue plasminogen activator (tPA), had a significant 3.9-fold increase in the IR-injured skeletal muscle (Figure 3a). To determine whether inhibition of PAI-1 would result in greater extracellular cleavage of proBDNF and a subsequent reduction in inflammation following hindlimb IR injury, wildtype animals were treated with the PAI-1 inhibitor, PAI-039 (tiplaxtinin) or the vehicle control [30]. Western blot revealed an increase in mature BDNF in the injured control limb of PAI-039-treated animals, and following IR injury, proBDNF expression had a 1.6-fold significant reduction in PAI-039-treated animals (Figure 3b). These data indicate that skeletal muscle proBDNF is cleaved extracellularly by plasmin. To determine whether reducing the level of proBDNF via PAI-039 in IR injury would affect the inflammatory phenotype, immunofluorescent staining for CD68—a proinflammatory macrophage marker—in the injured muscle was performed. In the vehicle controls following IR injury, there was a significant 1.2-fold increase in CD68+ staining; however, treatment with PAI-039 did not show any significant increase in CD68+ staining following IR injury (Figure 3c). Additionally, qPCR revealed a significant 1.3-fold decrease in proinflammatory cytokines IL-1B and a significant 9.4-fold reduction in CXCL1 in the injured tissue from animals treated with PAI-039 compared to vehicle-treated animals (Figure 3d). These data show that when proBDNF expression is reduced, the proinflammatory process is blunted in tourniquet-induced IR injury.

### 2.4. LM11A-31 Treatment Reduces Inflammatory Phenotype Following IR Injury

**Figure 4:**
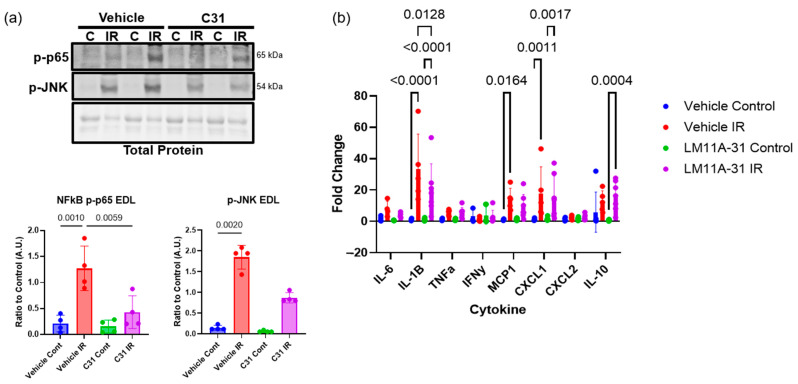
LM11A-31 treatment reduces inflammatory phenotype following IR injury. (**a**) Representative Western blots of phosphorylated (p) NFkB p65 and JNK in skeletal muscle from contralateral control (C) muscle tissue and IR-injured muscle tissue from vehicle- and LM11A-31 (C31)-treated animal and corresponding quantification (below); n = 4 analyzed using one-way ANOVA and Tukey’s post hoc test, significance at *p* < 0.05. (**b**) Quantification of qPCR for cytokines in vehicle- and C31-treated animals following IR injury represented as log fold changes; n = 6–7 analyzed using 2-way ANOVA followed by Tukey’s post hoc analysis, significance at *p* < 0.05. Error bars represent SD.

To test the role of proBDNF-p75^NTR^ signaling in IR injury, the small molecule LM11A-31 was used to modulate p75^NTR^ signaling during tourniquet-induced IR injury. Activation of the major p75^NTR^ pathways NF-κB and JNK and proinflammatory cytokine expression was assessed. Animals treated with LM11A-31 showed reduced p75^NTR^ signaling, as evidenced by a significant 3-fold reduction in phosphorylation of NF-κB and a significant 2.1-fold reduction in phosphorylation of JNK (Figure 4a), along with a significant 1.2-fold decrease in IL-1β and a 1.6-fold significant increase in anti-inflammatory IL-10 compared to vehicle-treated animals (Figure 4b). These data further suggest that the inflammatory phenotype following tourniquet-induced IR injury is driven at least partially by p75^NTR^ signaling.

### 2.5. p75^NTR^ Is Upregulated in Splenic Monocytes

**Figure 5:**
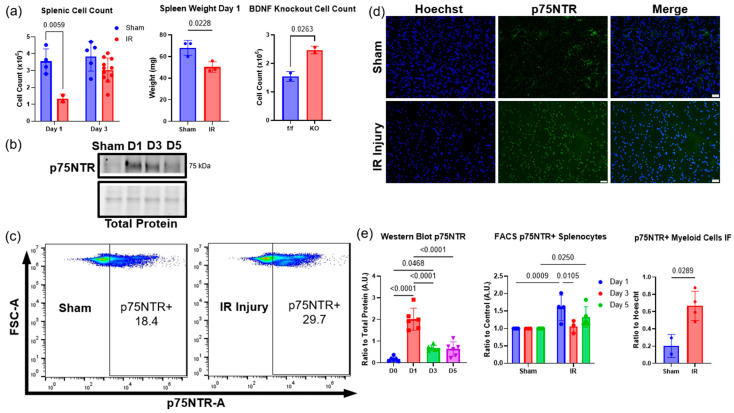
p75^NTR^ is upregulated in splenic monocyte populations. (**a**) Quantification of monocyte cells isolated from the spleen from sham and IR-injured animals on reperfusion days 1 and 3 (left); n = 2–10 analyzed using two-way ANOVA followed by Tukey’s post hoc test, significance at *p* < 0.05. Quantification of spleen weight on reperfusion day 1 in IR-injured animals compared to sham animals (middle); n = 3 analyzed using Student’s t test, significance at *p* < 0.05. Cell count on reperfusion day 1 in floxed control (f/f) and BDNF skeletal muscle knockout animals (KO); n = 2 analyzed using Student’s t test, significance at *p* < 0.05. (**b**) Representative Western blot of p75^NTR^ following sham or IR injury on reperfusion days 1, 3, and 5. (**c**) FACS analysis of p75^NTR^ expression in splenic monocytes isolated from sham and IR-injured animals on reperfusion days 1, 3 and 5. (**d**) Representative images of immunofluorescent staining for p75^NTR^ in sham and IR-injured animals. (**e**) Quantifications for Western Blot in panel (**b**) n = 6 analyzed using one-way ANOVA followed by Tukey’s post hoc test, significance at *p* < 0.05 (left); quantification of splenic monocytes FACS in panel (**c**) n = 2–4 analyzed using two-way ANOVA followed by Tukey’s post hoc test, significance at *p* < 0.05 (middle); and quantification for immunofluorescent staining in panel (**d**) n = 2–4 analyzed using Student’s *t* test, significance at *p* < 0.05 (right). Error bars represent SD.

Compared to sham animals, the spleen weight was significantly reduced 1.4-fold and the total splenic monocytes of animals with IR injury were significantly reduced 2-fold, indicating increased monocyte recruitment from the spleen to the site of injury, which is consistent with previous publications [26,28] (Figure 5a). Additionally, in BDNF skmKO animals, the spleen had a significant 1.6-fold increase in monocytes compared to the flox control mice (Figure 5a), suggesting that skeletal muscle-derived proBDNF may be responsible for driving migration of splenic monocytes to the site of injury. Using Western blot, we showed that the receptor for proBDNF, p75^NTR^, had a 3.2-fold significant increase in expression in splenic monocytes isolated on reperfusion day 1 of tourniquet-induced IR injury (Figure 5b). Flow cytometry analysis showed an increase in p75^NTR^+ in monocytes isolated from animals with IR injury, which confirmed the Western blot results (Figure 5c). Using immunofluorescent staining for p75^NTR^ in monocytes isolated from sham or IR-injured animals revealed that IR-injured animals showed a 3.4-fold significant increase in p75^NTR^ (Figure 5d). Although we isolated splenic myeloid cells, some lymphocytes may still be present; therefore, we also stained for p75^NTR^ in lymphocytes using CD3+ and CD4+ makers for T cell populations. We did not see any change in p75^NTR^ expression between sham and IR-injured animals in these populations confirming that p75NTR expression is increased in the myeloid cell populations specifically.

### 2.6. IR Injury Alters Innate Immune Response to LPS

**Figure 6:**
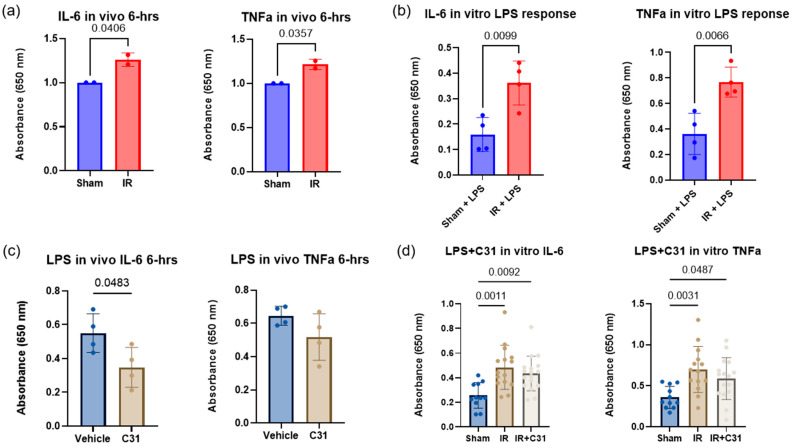
IR injury alters the innate immune response to LPS. (**a**) Quantification showing results from ELISA comparing sham and IR-injured animals, looking at IL-6 and TNF-α at 6 h post in vivo IP LPS injection on reperfusion day 3; n = 2 analyzed using Student’s t test, significance at *p* < 0.05. (**b**) Quantification showing results from ELISA comparing monocytes isolated from sham and IR-injured animals on reperfusion day 3, looking at IL-6 and TNF-α after in vitro LPS stimulation; n = 4 analyzed using Student’s t test, significance at *p* < 0.05. (**c**) Quantification showing results from ELISA comparing LM11A-31 (C31) and vehicle-treated animals, looking at IL-6 and TNF-α at 6 h post in vivo LPS injection; n = 4 analyzed using Student’s t test, significance at *p* < 0.05. (**d**) Quantification showing results from ELISA comparing monocytes isolated from sham or IR-injured animals treated with C31 on reperfusion day 3, looking at IL-6 and TNF-α after in vitro LPS stimulation; n = 10–16 analyzed using one way ANOVA followed by Tukey’s post hoc test, significance at *p* < 0.05. Error bars represent SD.

Following either tourniquet-induced IR injury or sham procedure, animals were given lipopolysaccharide (LPS), a bacterial endotoxin, via IP injection (5 mg/kg) on reperfusion day 3. The results show that, compared to sham animals, animals with IR injury had significantly higher innate immune responses to LPS, as indicated by increased levels of IL-6 (1.3-fold increase) and TNF-α (1.2-fold increase) in the serum measured by ELISA 6 h post LPS injection (Figure 6a). To test whether this increased innate response to LPS in IR-injured animals could be attributed to the splenic monocyte populations, splenic monocytes were isolated and stimulated with LPS in vitro for 3 h. IL-6 and TNF-α levels in the media were measured using ELISA, and the results mirrored the in vivo findings, showing that monocytes isolated from IR-injured animals were more reactive to LPS than monocytes from sham animals (Figure 6b). These data show that tourniquet-induced IR injury primes the innate immune system, leading to stronger responses to secondary challenges.

### 2.7. p75^NTR^ Is Involved in Splenocyte Innate Immune Function

To test whether the increased immune response to LPS was due to signaling through the upregulated p75^NTR^, animals were pretreated with LM11A-31 or the vehicle control for three days prior to IP injection of LPS. Interestingly, the response to LPS in LM11A-31-treated animals showed a 1.6-fold significant reduction in IL-6 compared to vehicle-treated animals (Figure 6c). Additionally, monocytes isolated from sham, vehicle-treated, IR-injured, or LM11A-31-treated IR-injured animals were treated with LPS in vitro. The results showed a trend toward reduced IL-6 and TNF-α in the cells from LM11A-31-treated animals (Figure 6d). Since modulating p75^NTR^ signaling via LM11A-31 reduced the inflammatory response to LPS, these results suggests that p75^NTR^ signaling may play a role in modulating the innate inflammatory response.

## 3. Discussion

ProBDNF has been established as an important neurotrophic factor in the central nervous system, but the importance of proBDNF in skeletal muscle is still being unveiled [4,5,6,31,32]. In this study, we show strong evidence to suggest that proBDNF is not only the dominant form of BDNF expressed by skeletal muscle, but that proBDNF is released into circulation from IR-injured skeletal muscle, exerting global effects such as systemic inflammation.

We previously showed that proBDNF modulates inflammation in skeletal muscle following tourniquet-induced IR injury [20,21]; however, the question of how proBDNF modulated the proinflammatory response remained. Using a mouse model that allowed for specific protein labeling in skeletal muscle and a mouse model of skeletal muscle-specific knockout of BDNF, this study clearly demonstrates that the predominant source of the increased serum proBDNF is the injured skeletal muscle. This is very convincing evidence supporting the idea that proBDNF is a myokine. A series of functional tests in this study show that muscle-released proBDNF is involved in local and systemic inflammatory responses following muscle injury. These results advance our understanding of myokine proBDNF functioning not only in a paracrine but also in an endocrine manner.

We previously reported that BDNF skmKO animals subjected to tourniquet-induced IR injury or sciatic denervation showed a reduced proinflammatory phenotype [20,21]. We further confirm these results here and show that in the absence of proBDNF-driven inflammation, skeletal muscle functional recovery was slowed. We chose to perform this analysis on day 12 based on time course studies performed in our lab (data unpublished) on wildtype animals that showed that by 12–14 days post-injury, full functional recovery was achieved. The delay in functional recovery seen in BDNF skmKO animals was not surprising, as it has been shown that acute inflammation is a necessary component of skeletal muscle regeneration and functional recovery. However, this is a novel way to show the importance of proBDNF in promoting the function recovery of injured skeletal muscle.

The cleavage of proBDNF to mature BDNF has been shown to occur both intracellularly and extracellularly [23,32]. One protein responsible for the extracellular cleavage of proBDNF is plasmin [33,34]. For plasmin to be active, its precursor, plasminogen, must first be cleaved by tissue plasminogen activator (tPA), which is negatively regulated by plasminogen activator inhibitor (PAI-1) [30,33,34]. Here, we show that IR injury increased expression of PAI-1, suggesting a potential cause of the higher levels of proBDNF released from skeletal muscle following IR injury. By inhibiting PAI-1 pharmacologically using PAI-039, proBDNF was reduced following IR injury. This confirms that proBDNF is a substrate for tPA; importantly, it also confirms that pharmacologically reducing proBDNF results in a similar reduction in inflammation as seen in skeletal muscle knockout animals. This is strong evidence supporting proBDNF as a driver of inflammation following skeletal muscle injury.

Showing that proBDNF can be released into circulation to participate as an endocrine signaling molecule drives the question of what the target tissue or cell type is for the circulating proBDNF. The present study shows the proBDNF receptor p75^NTR^ is significantly upregulated on splenic monocyte populations. Knowing that these cells are capable of being quickly recruited to the site of injury and seeing that p75^NTR^ is significantly upregulated makes these monocyte populations a very likely target for circulating proBDNF. In addition to p75^NTR^ being significantly upregulated on monocytes following IR injury, these monocytes exhibited enhanced inflammatory response to LPS stimulation, which is attenuated by the treatment with LM11A-31. Together, these results indicate that the circulating proBDNF released from the injured muscle targets the upregulated p75^NTR^ in monocytes to promote the inflammatory response. While not all proinflammatory markers were reduced in BDNF skmKO-, PAI-039-, or LM11A-31-treated animals compared to controls, we attribute this to the specific inflammatory process regulated by proBDNF-p75^NTR^ signaling. While we have shown this signaling pathway is significant to the inflammatory process following tourniquet-induced IR injury, inflammation requires a complex coordination of events and thus it is not the only pathway involved. We have observed that proBDNF treatments in monocytes isolated following IR injury did not cause increased cytokine release and migration, suggesting that proBDNF is unlikely to function as a damage-associated molecule pattern (DAMP) that can directly cause an inflammatory response. Instead, the proBDNF-p75^NTR^ signaling pathway may enhance the inflammatory responses via priming or increasing expression of pattern recognition receptors (PRRs) such as toll-like receptors (TLRs). LPS is a potent stimulant that induces innate immune responses via TLR4. Thus, showing that IR injury increases the responsiveness of monocytes to LPS and that, by modulating p75^NTR^ signaling with LM11A-31, the response to LPS was reduced, we show strong, novel evidence that proBDNF-p75^NTR^ signaling may regulate the proinflammatory pathways via changes in PRR expression. Our data suggest that proBDNF and p75^NTR^ are promising novel therapeutic targets to control injury-induced inflammation and immune activities.

LM11A-31 is a well-known small-molecule modulator of p75^NTR^ and has been extensively studied in various neuronal disorders and injuries. Preclinical studies showed that LM11A-31 reduces amyloid-induced synapse loss and cognitive deficits [35,36] and inhibits a wide range of tau molecular pathologies and their sequelae in P301S tauopathy mice [37]. The recently published results of a randomized double-blind, placebo-controlled phase II clinical trial show evidence that LM11A-31 treatment slows the progression of pathophysiological features of Alzheimer’s disease [38]. The results of this study reveal the role of altered p75^NTR^ signals in immune cells such as monocytes in sterile inflammation, which can help us to better understand the potential mechanism underlying the beneficial effects of LM11A-31 in neurodegenerative diseases.

While the greatest increase in serum proBDNF was seen on reperfusion day 1, experiments looking at inflammatory markers were performed based on our previous studies, in which we saw that the inflammatory markers had the greatest increase on reperfusion day 3.

While proBDNF plays a substantial role in the local proinflammatory response following skeletal muscle injury, it is important to understand its impact on injury healing and tissue recovery. The present data suggest that proBDNF and the proinflammatory response it produces are necessary for promoting the regeneration phase following skeletal muscle injury. This is not a surprising finding, since it is known that acute inflammation is a necessary process for removing damaged tissue and promoting healing and recovery from injury or disease. It is also well known that timely inflammation resolution is critical for tissue recovery and regeneration. Therefore, it would be interesting and clinically important to examine whether delayed inhibition of proBDNF-mediated inflammation after the acute phase of injury may positively affect the injury recovery. Interestingly, we observed that while treatment with LM11A-31, a modulator of p75^NTR^, decreased inflammation in injured muscle, it has no significant negative effect on contraction performance in normal muscles. Therefore, treatments with LM11A-31 at appropriate time points targeting upregulated proBDNF-p75^NTR^ signaling may provide a novel approach for treating injury-induced inflammation without compromising muscle recovery.

## 4. Materials and Methods

### 4.1. Animal Models

All experimental protocols and the use of animals in this study were reviewed and approved by the University of South Dakota Institutional Animal Care and Use Committee (IACUC) according to NIH guidelines on the care and use of animals in research. Male and female C57BL/6J mice chosen at random at 12–16 weeks of age were used for all experiments.

### 4.2. Skeletal Muscle-Specific BDNF Knockout Animals

C57BL/6J mice with the *BDNF* gene coding region flanked by loxP sites (BDNF2lox) were purchased from Jackson Labs (strain #: 033689) (Bar Harbor, ME, USA) [39,40]. Homozygous *BDNF* floxed mice were crossed with mice expressing Cre recombinase promoted by either the muscle-specific myosin light-chain polypeptide 1 promoter (*Myl1*) (strain #: 024713) or the human alpha-skeletal actin promoter (HSA79) (strain #: 006149) (Bar Harbor, ME, USA) [41,42,43].

### 4.3. Skeletal Muscle Mutant MetRS* Knock-In Animals

C57BL/6J mice expressing the ROSA26 locus knock-in STOPflox GFP-R26-MetRS* mutation, a point mutation in the methionyl-tRNA synthetase gene, L274G, were purchased from Jackson Labs (strain #: 028071) (Bar Harbor, ME, USA) [29]. Homozygous mice were obtained by backcrossing the F1 generation, then were crossed with mice expressing Cre recombinase promoted by the muscle-specific human alpha-skeletal actin promoter (HSA79) (strain #: 006149) (Bar Harbor, ME, USA) to produce mice with skeletal muscle-specific expression of the modified methionyl-tRNA [43]. In this way, the noncanonical amino acid azidonorleucine, a methionine analog, when given to mice via IP injection (100 mM of ANL in PBS, ip 10 mL/kg daily for 1 week), can be incorporated into nascent amino acids only in the muscle tissue expressing the mutant tRNA synthetase [29,42]. The expression of MetRS* was confirmed by the presence of GFP in skeletal muscle tissue using Western blot.

### 4.4. Genotyping

For genotyping of knockout and transgenic animals, Promega GoTaq green master mix (M7122) was used to combine forward and reverse primers for *BDNF*, *Cre*, 6 Gt mutant, or 6 Gt wildtype were used. PCR reactions were run in an Eppendorf Mastercycler Nexus gradient PCR thermocycler (Ocala, FL, USA). The PCR products were then run out on a 2% agarose gel containing ethidium bromide for nucleic acid staining at 100 V in 1× TBE buffer for 1 h alongside a 1000 bp ladder. Gels were then imaged on a Syngene Ingenius gel documentation system (Hampton, NH, USA).

### 4.5. Hindlimb Ischemia–Reperfusion Injury Model

Mice were subjected to acute ischemia–reperfusion (IR) injury while anesthetized using 1.5–3% isoflurane gas on a water pad heating pad set at 42 °C. To achieve complete ischemia in the right hindlimb, an orthodontic rubber band was placed at the level of the hip using a McGivney ligator applicator (Jersey City, NJ, USA), leaving the left hind limb as a contralateral control [10,21,44,45]. Complete ischemia was confirmed using laser Doppler imaging (Moor Instruments) (Wilmington, DE, USA) in which the perfusion of the limb could be assessed [21]. During the ischemic period, buprenorphine SR was administered subcutaneously at 1 mg/kg to control pain. Following 1–2 h of ischemia, the orthodontic rubber band was removed, allowing for reperfusion of the limb for 1–12 days [21]. Following the reperfusion period, animals were sacrificed, and tissue was collected from the IR-injured and contralateral control limbs [21].

### 4.6. In Vivo LPS Injection

Stock *E. coli* LPS solution (20 mg/mL) (St. Louis, MO, USA) was diluted to a working solution concentration of 5 mg/mL. Animals were given 5 mg/kg by IP injection of 0.1 mL/10 g of body weight of working solution. At 2 and 6 h post-injection, 20 μL of blood was collected from the tail by clipping the end. The collected blood was added to 40 μL of PBS and centrifuged at 1000× *g* for 10 min to collect the serum. The collected serum was then used for ELISA.

### 4.7. Treatment with LM11A-31

LM11A-31 was provided by our collaborator, Dr. Frank Longo at Stanford University, who previously tested the compound for chemical identity and at >99% purity. The compound was dissolved in deionized water to a concentration of 50 mg/mL, aliquoted in 1.5 mL tubes, and stored at −20 °C until use. As an orally active compound, for treatment, 1 mL of stock solution was diluted 10-fold by placing it in 9 mL of drinking water. Animals were then gavage-fed the compound or the vehicle (drinking water) at a rate of 50 mg/kg/day beginning three days prior to undergoing IR injury and throughout the reperfusion period [20,21,36,46,47,48,49].

### 4.8. PAI-039 Treatment

PAI-039 (tiplaxtinin) (Selleckchem, Huston, TX, USA) was dissolved in DMSO to 2 mg/mL concentration. The stock solution was then placed in drinking water at a concentration of 12 μg/mL (2 mg/kg/day) based on the water consumption rate of 5 mL of water per mouse per day [30]. Wildtype C57BL/6J mice were given PAI-039 (tiplaxtinin) treatment in drinking water or the vehicle control beginning three days prior to undergoing hindlimb IR injury, and until reperfusion day 3 when the tissue was collected for analysis. No significant differences were seen in the water consumption rate between the two groups.

### 4.9. In Situ Contraction

Animals were anesthetized using an IP injection of 0.1 mL/10 g of body weight of urethane (2 g/kg) and α-chloralose (5 mg/kg) solution. After confirming anesthetization by a lack of pain response, mice were placed in the prone position on a 42 °C hot water warming pad. A cut was carefully made near the distal tendon of the gastrocnemius muscle and skin was removed, taking care not to damage underlying muscle tissue. A small piece of paper towel was wetted in prewarmed PBS and placed atop the exposed muscle to prevent it from dying. A piece of 4.0 suture was placed under the distant tendon of the gastrocnemius and a copper electrode was inserted into the tendon and pushed up into the muscle. The suture was then tied with a triple knot around the tendon and electrode, leaving a loop to attach the force transducer, before cutting the tendon and freeing the distal end of the muscle. The proximal end of the soleus muscle was then freed and placed in a 1.5 mL tube for Western blot analysis along with the EDL. A silver reference electrode was placed under the proximal end of the gastrocnemius muscle and secured in place with tape. A thick gauge metal wire was placed across the distal end of the tibia and taped down on either side of the limb to prevent the leg from changing position, along with a retractor on the hip stretched across the body to further stabilize the limb. The transducer was then hooked through the loop left in the suture and adjusted to ensure a right angle between the transducer and direction of muscle contraction.

With the PowerLab, stimulator, and charting software turned on and calibrated, the pretest was performed. The stimulator was set to a duration of 2 ms, 10 V, 100 Hz, a training rate of 1 TPS, and a training duration of 300 ms. The transducer was tightened to gently stretch the muscle to preload the muscle to 2 g of force.

For the preload–force test, presets were held at 2 ms, 10 V, and 100 Hz and the contractile force was measured with preload at 2 g, 4 g, 6 g, 8 g, and 10 g. For frequency, presets were held at 2 ms, 6 g preload, and 10 V, and contraction was measured at 20, 40, 60, 80, 100, and 150 Hz. For amplitude force, the presets were 2 ms, 6 g, and 100 Hz, and contraction was measured at amplitudes of 1, 2, 4, 6, 8, and 10 V. All tests were performed on the injured and control limb, then gastrocnemius muscles were weighed.

### 4.10. Tissue Collection

Animals were anesthetized using 3% isoflurane or with an IP injection of 0.1 mL/10 g of body weight of urethane (2 g/kg) and α-chloralose (5 mg/kg) solution. For serum collection, after confirming anesthetization by a lack of pain response, mice were decapitated, and blood was collected in a 1.5 mL tube. Blood was coagulated at 4 °C for 30 min or more and then centrifuged at 1000× *g* for 10 min. The serum was transferred to a new 1.5 mL tube and stored at −80 °C. For collection of the hindlimb muscles, with the mouse in the prone position, a small incision was made in the skin at the distal tendon of the gastrocnemius muscle using blunt-tip scissors. The skin was then retracted and cut away to reveal the underlying muscle groups.

For Western blot, the soleus and EDL were first carefully removed using forceps to isolate the muscle and scissors to cut the distal and proximal tendons. The muscles from both the injured and uninjured control limbs were placed in a prechilled 1.5 mL tube on dry ice. Next, the plantaris was removed similarly for qPCR, followed by the gastrocnemius muscle, which was used for immune cell isolation for flow cytometry, or placed on ice. Lastly, the tibialis anterior was isolated, coated in OCT, and forceps dipped in prechilled 2-methylbutane on dry ice were used to flash freeze the tissue for histological and immunofluorescent staining. The brain and heart were processed similarly for Western blot and PCR analysis.

### 4.11. Splenic Monocyte Isolation

Mice were anesthetized using IP injection of 0.1 mL/10 g of body weight of urethane (2 g/kg) and α-chloralose (5 mg/kg) solution. After anesthetization was confirmed, the mice were decapitated, and blood was collected in 1.5 mL tube for serum collection as described above. The rest of the protocol was adapted from BD Sciences Preparation of Murine Splenocytes (Franklin Lakes, NJ, USA) [50]. The mouse was placed in the supine position, and the skin was saturated with 70% ethanol. Then, using sterile forceps, the skin was grasped, and an incision was made to access the upper abdominal cavity. The spleen was located and carefully removed. It was then placed in a 35 mm pre-weighed Petri dish with 100 μL of DMEM and then weighed. In a tissue culture hood, the spleen was crushed using a sterile 0.5 mL test tube lid. The splenocytes were collected in 2 mL of DMEM then strained through a 70 μm cell strainer into a 50 mL tube. The remaining spleen tissue was crushed once more in 100 μL of DMEM, collected in 2 mL of DMEM, and added to the strainer. Using 5 mL of DMEM, the cells were washed through the strainer, then collected cells were centrifuged at 1600 rpm for 5 min. The supernatant was aspirated, and cells were resuspended in 2 mL of red blood cell lysis buffer and incubated in a 37 °C water bath for 2 min. Following the incubation period, 10 mL of DMEM was added to stop the lysis and cells were again collected by centrifugation at 1600 rpm for 5 min. After aspirating the supernatant, cells were resuspended in 10 mL of DMEM and 5 mL were placed into two 70 mm Petri dishes. Dishes were placed in a cell incubator at 37 °C and 5% CO_2_ for 1 h to allow monocytes to adhere. Following the incubation period, cells were gently swirled to remove nonadherent cells, and the media was aspirated. After one more wash with fresh DMEM, adherent cells were collected by forcefully pipetting media and collecting media containing cells into a 50 mL tube. Total volume was increased to 16 mL using DMEM then cells were counted and processed for further experimentation.

### 4.12. In Vitro LPS-Induced Cytokine Release

Stock *E. coli* LPS solution (20 mg/mL) was diluted to a working solution concentration of 20 μg/mL in 1 mL of DMEM. Isolated splenic monocytes were placed into 35 mm Petri dishes or 1.5 mL test tubes and 100 ng/mL of LPS was added. Following incubation for 3 h at 37 °C, cells were collected and centrifuged at 10,000 rpm for 10 min, and 100 μL of supernatant was added to ELISA plates.

### 4.13. In Vitro LM11A-31 Treatment

LM11A-31 stock solution (50 mg/mL) was diluted using DMEM and was added to cells at a concentration of 100 ng/mL. For LPS stimulation, LM11A-31 was added 1 h prior to treatment.

### 4.14. Trans-Well Migration Assay

The trans-well assay procedure was adapted from that described by Justus et al. [50,51,52,53]. Isolated splenic monocytes were normalized to a concentration of 10 × 10^6^ cells/mL in migration media and 100 μL of cells were added to the trans-well insert and incubated for 10 min to allow the cells to settle. Following incubation, 600 μL of migration media (control) or migration media containing 200 ng/mL proBDNF were added to each well and cells were allowed to migrate for 4 h. Following migration, trans-wells were removed from the media, and cotton swabs were used to clear unmigrated cells and media from the top of the trans-well membrane. Cells were fixed in 1 mL of 70% ethanol for 15 min at room temperature. Following fixation, the trans-wells were allowed to air dry prior to staining using Hoechst (1:1000 dilution) for 10 min at room temperature. Cells were then washed three times by placing trans-wells in 24-well plates containing PBS and imaged using a fluorescence microscope. Three images were taken per well. To analyze the data, images were opened in ImageJ, color channels split, the threshold was set, and particles were analyzed.

### 4.15. Western Blot

Western blot was performed as described in our previous publications [20,21,54,55]. Briefly, soleus, EDL, and brain samples were collected, snap frozen, and held at −80 °C until they were processed. Samples were lysed in 1× RIPA buffer supplemented with phosphatase inhibitor cocktail, protease inhibitor cocktail, and proteasome inhibitor MG132 using pestles in 1.5 mL tubes. Protein concentrations of the samples were equalized as determined using a Pierce BCA assay. Protein samples were subjected to standard SDS-PAGE electrophoresis and blotted using primary antibodies. Western blots were visualized using appropriate secondary antibodies conjugated with fluorescent dyes and LiCor scanner. For loading controls, total protein imaging was used by incorporation of 2,2,2-trichloroethanol into the gels and then imaging the gel for protein bands prior to the transfer step of Western blot protocol [56].

### 4.16. qPCR

The plantaris muscles from both the IR and control limbs were collected and homogenized using pestles in 1.5 mL test tubes containing 400 μL of RNA-zol. Samples were processed according to the manufacturer’s instructions (Zymo Research Direct-zol RNA miniprep kit (Irving, CA, USA)). The final RNA product was analyzed for purity and concentration using Thermo Scientific Nanodrop 2000 (Waltham, MA, USA). RNA was then transcribed into cDNA using Applied Biosystems Multiscribe high-capacity cDNA kit (Waltham, MA, USA) according to the manufacturer’s instructions. The cDNA was then used in conventional PCR using Promega GoTaq master mix (Madison, WI, USA).

### 4.17. ELISA

ELISA assays were performed with 10 μL of whole muscle lysate from the soleus muscles of IR and contralateral control limbs lysed with 1× RiPA buffer supplemented with phosphatase inhibitor cocktail, protease inhibitor cocktail, and proteasome inhibitor MG132 according to the manufacturer’s instructions (Biolegend ELISA Max (San Diago, CA, USA)). The IR limb was normalized to the control limb and the data were analyzed over the time course of IR [21]. For serum samples, 50 μL of serum was diluted with 50 μL of assay dilutant. For media, 100 μL of media were used undiluted.

### 4.18. Flow Cytometry

Cells were collected and spun down at 900× *g* for 2 min. The supernatant was aspirated, and cells were resuspended in 200 μL of FACS buffer to wash the cells. Cells were again centrifuged at 900× *g* for 2 min and supernatant was aspirated. Cells were then resuspended in Fc Block (TruStain FcX PLUS (156603) (Biolegend, San Diego, CA, USA) at a rate of 0.25 μg/106 cells and incubated on ice for 10 min. Cells were again collected by centrifugation and the supernatant was aspirated and resuspended in FACS buffer to wash, then centrifuged. Following aspiration of the supernatant, cells were again resuspended in FACS buffer and divided into test tubes for staining or negative control (50 μL total volume per tube). Then, 50 μL of 2x fluorescently conjugated antibody cocktail was added to the samples for extracellular staining/makers and incubated on ice while protected from light for 30 min.

For intracellular staining, following staining for extracellular markers, the cells were resuspended in 500 μL of fixation buffer and incubated for 10 min at RT, vertexing occasionally to prevent clumping. The cells were spun down at 300× *g* for 2 min, supernatant was aspirated, and the cells were washed twice with FACS buffer. Following the final wash, cells were resuspended in 50 μL of permeabilization buffer and 50 μL of 2× fluorescently conjugated intracellular antibody cocktail was added and the cells were incubated on ice for 30 min. After incubation, cells were spun down (900× *g* for extracellular or 300× *g* for intracellular staining) and washed twice with FACS buffer. After the final wash, the cells were resuspended in 200 μL FACS buffer and added to a 5 mL FACS tube with 300 μL FACS buffer. For flow cytometry analysis, the samples were analyzed using the BD Accuri C6 flow cytometer (Franklin Lakes, NJ, USA). Data were analyzed using FlowJo software version 10.8.1(BD Biosciences, Franklin Lakes, NJ, USA).

### 4.19. Immunofluorescent Staining

Sections were cut to 10 um using a Leica cryostat (Nussloch, Germany) set at −18 °C and placed on charged slides. Frozen muscle sections were washed at room temperature in PBST for 5 min, fixed with 4% paraformaldehyde (PFA) for 10 min, and rinsed with PBST again for 5 min. Sections were microwaved in 10% tri-sodium citrate (pH 6.0) for 20 min, maintaining a temperature of 90–100 °C. After 20 min, slides were allowed to cool to room temperature in the tri-sodium citrate, followed by a 3 × 5 min PBST wash. The sections were then incubated with primary antibodies at 4 °C overnight. Following incubation, slides were washed in PBS 3 × 5 min, mounted with coverslips using Fluoromount mounting solution, and imaged using a Confocal (Olympus Fluoview 500, Center Valley, PA, USA) or Olympus D microscope (Breinigsville, PA, USA) [21].

### 4.20. H&E Staining

Slides were taken from the −80 °C freezer and allowed to adjust to room temperature. Slides were then placed in Meyer’s hematoxylin solution in a staining jar for 10 min to stain the nuclei. The slides were transferred to a staining jar with running tap water until the water was clear. Next the slides were placed in a staining jar with Eosin solution for 3 min, then washed under running tap water again. Slides were then dehydrated by successively transferring the slides into staining jars with 70% ethanol for 20 s, 90% ethanol for 20 s, 100% ethanol for 1 min, and xylene for 3 min. The slides were then allowed to dry, and they were mounted with toluene-based mounting media and covered with cover slides.

### 4.21. Statistical Analysis

Data are presented as mean ± standard deviation (SD). Following collection, data were analyzed for normal distributions and Rout’s test for outliers was performed. Student’s *t* test, one-way ANOVA, or two-way ANOVA were then used, followed by Tukey’s post hoc test; specific uses are indicated in the Figure legends. Statistics were analyzed using GraphPad Prism 10 software. Differences were considered statistically significant at *p* < 0.05 [21].

## 5. Conclusions

Increased release of proBDNF from injured skeletal muscle stimulates upregulated p75^NTR^ in peripheral monocytes to promote injury-induced inflammation. Treatment with p75^NTR^ modulator LM11A-31 offers a novel therapeutic against inflammation in muscle injuries and other potential pathological conditions.

## Data Availability

All original raw data related to this study are saved in the USD BBS research shared drive and are available upon request.
